# Exercise Alleviates Osteoporosis and Hyperglycemia in Type 1 Diabetes Mellitus Mice via Piezo1-Mediated Mechanotransduction

**DOI:** 10.3390/biology15110819

**Published:** 2026-05-22

**Authors:** Mengshu Cao, Fang Pang, Yanan Yu, Junzheng Yu, Sainan Ma, Lijun Sun, Xiushan Fan, Liang Tang

**Affiliations:** Institute of Sports Biology, Physical Education School, Shaanxi Normal University, Chang’an Campus, Xi’an 710119, China; caomengshu@snnu.edu.cn (M.C.);

**Keywords:** exercise, type 1 diabetes mellitus, osteoporosis, hyperglycemia, Piezo1

## Abstract

Osteoporosis and hyperglycemia are increasingly recognized as dual public health concerns in T1DM. However, the precise molecular mechanisms by which exercise ameliorates these conditions, particularly the contribution of mechanosensitive channels such as Piezo1, remain incompletely elucidated. To explore these mechanisms, T1DM mice were subjected to a 6-week treadmill training protocol (15 m/min, 20 min/day, 6 days/week) to evaluate the functions of exercise on diabetic osteoporosis and hyperglycemia. Exercise intervention markedly improved bone quality in T1DM mice, alleviating osteoporotic manifestations, as evidenced by enhanced mechanical strength, restored bone microarchitecture, and normalized histomorphology. Concurrently, exercise significantly reduced hyperglycemia. To clarify the role of Piezo1, mechanical stretch was applied to *Piezo1*-knockout MC3T3-E1 (*Piezo1*^−/−^) cells in vitro, mimicking the mechanical stimulation induced by exercise. Consistent with the in vivo results, mechanical stimulation facilitated osteogenic differentiation and glucose metabolism through Piezo1-mediated mechanotransduction. Importantly, these beneficial effects were substantially abrogated in *Piezo1*^−/−^ cells, highlighting the central role of Piezo1. Collectively, these findings demonstrate that Piezo1-mediated mechanotransduction constitutes a critical factor by which exercise mitigates osteoporosis and hyperglycemia in T1DM mice. This study provides a framework for the development of new therapeutic strategies targeting Piezo1-mediated mechanotransduction for T1DM management.

## Simple Summary

Osteoporosis and hyperglycemia are major public health concerns in type 1 diabetes mellitus (T1DM); nevertheless, the mechanisms through which exercise alleviates these complications remain inadequately elucidated. In this study, we employed a 6-week treadmill exercise protocol in a T1DM mouse model to investigate the role of the mechanosensitive ion channel Piezo1 in mediating these therapeutic effects. Exercise improved bone mechanical strength, microarchitecture, and histomorphology while concurrently reducing hyperglycemia. In vitro, mechanical stimulation facilitated osteogenic differentiation and glucose metabolism in MC3T3-E1 cells, effects that were abrogated in *Piezo1*-knockout cells. These results demonstrate the critical involvement of Piezo1-mediated mechanotransduction in the osteogenic and glycemic improvements induced by exercise in T1DM, positioning Piezo1 as a promising therapeutic target.

## 1. Introduction

Diabetic osteoporosis is a chronic, systemic metabolic bone disorder characterized by an elevated risk of osteoporotic fractures, resulting from dysregulated glucose metabolism, accumulation of advanced glycation end products, and osteovascular impairment [1,2]. Furthermore, diabetic osteoporosis represents one of the most prevalent and earliest-onset complications of T1DM [3]. Although T1DM is often regarded as a childhood disease, about 50% of new diagnoses occur in adults [4]. The global incidence of T1DM is increasing at an annual rate of 2–3% [5]. These trends underscore the urgent need for targeted interventions and preventive strategies to mitigate the burden of diabetic osteoporosis.

Although osteoporosis and hyperglycemia in T1DM are increasingly recognized as coexisting health challenges, most therapeutic strategies address these conditions separately. Traditionally, various approaches have been employed to manage osteoporosis [6,7,8,9,10,11] and hyperglycemia [12,13,14,15,16,17,18,19]. However, these interventions are constrained by notable limitations, including adverse effects, high costs, and the inherent difficulty of targeting two distinct pathogenic pathways simultaneously [6,7,12,20,21]. Exercise, as a widely accessible non-pharmacological intervention, represents a central strategy for the prevention of both diabetes and osteoporosis [22,23], while also enhancing metabolic regulation in T1DM patients with residual pancreatic β-cell function [24,25]. Its systemic anti-inflammatory properties may further mitigate autoimmune pathogenesis [26], providing a strong rationale for the recommending exercise in T1DM management due to its combined metabolic and immunomodulatory effects. Nevertheless, existing research has predominantly focused on glycemic control, with limited evidence regarding its impact on T1DM-associated osteoporosis. Critically, a shared molecular mediator linking exercise to both enhanced bone anabolism and systemic glucose homeostasis in T1DM has yet to be identified.

Elucidating the molecular mechanisms underlying these comorbidities is crucial for the development of effective therapeutic strategies. Piezo1, a mechanosensitive ion channel, concerts mechanical stimuli into biochemical signals and plays a critical role in maintaining bone homeostasis [27,28]. As a sensor of shear stress, Piezo1 provides a potential molecular framework for understanding how exercise—a primary mechanical stimulus—may simultaneously improve both bone integrity and glucose metabolism in T1DM.

Although exercise is a well-established non-pharmacological intervention for alleviating hyperglycemia and improving bone health, the precise molecular mechanisms by which mechanical loading during exercise exerts therapeutic effects in the context of diabetic osteoporosis remain largely undefined. Recent advances have identified Piezo1 as a critical mechanosensor in bone cells; however, its role in the pathological interplay between diabetic bone fragility and impaired glucose metabolism has not been elucidated. This study advances the field by positioning Piezo1 as the central mechanobiological mediator orchestrating the concurrent amelioration of skeletal and metabolic complications. Beyond documenting symptomatic improvements, we provide evidence for a Piezo1-dependent “skeletal–metabolic axis” responsive to exercise. This conceptual framework not only enhances our understanding of exercise physiology but also highlights the potential of targeting Piezo1 with mechano-mimetic therapeutics. Such strategies could replicate the multi-systemic benefits of exercise in diabetic patients with severe mobility limitations, representing a transformative approach in the management of diabetic complications. This work aims to elucidate the mechanistic link between exercise and improved bone health and glucose homeostasis in T1DM mice.

## 2. Materials and Methods

### 2.1. Animals

Thirty-two male C57BL/6J mice (7-weeks-old) were provided by the Experimental Animal Center of Xi’an Jiaotong University (Xi’an, China). The mice were housed in a controlled environment with a 12 h light/dark cycle, a temperature range of 22–25 °C, and a relative humidity of 60 ± 5%. All experimental procedures were conducted in accordance with the National Institute of Health Laboratory Animal Care and Use Guidelines (NIH Publication No. 8023, revised in 1978) and were approved by the Animal Ethics Committee of Shaanxi Normal University (Approval No. 202316023). The animals were given free access to water and standard feed throughout the experiment.

### 2.2. T1DM Induction and Exercise Intervention

After a one-week acclimation period, all the mice were randomly divided into four groups (*n* = 8): normal control (NC), diabetic model mice (DM), diabetic mice treated with insulin (DM+INS), and diabetic mice subjected to exercise (DM+EX). For five consecutive days, the diabetic groups were administered daily intraperitoneal injections of streptozotocin (STZ, Sigma-Aldrich, Cat. No. 18883664, St. Louis, MO, USA; dissolved in 0.1 M citrate buffer; 60 mg/kg/day). To minimize the risk of false-positive results, the NC group was injected with an equivalent volume of sodium citrate buffer. The induction of T1DM mice was verified by collecting tail vein blood samples to measure fasting blood glucose (FBG) levels on days 5, 7, 10, and 14 following STZ injection. Throughout the study, weight, FBG, food intake, and water intake were monitored weekly. The mice (DM+INS group) received a daily subcutaneous insulin injection of 0.5–1 U at a consistent time each day. Over a six-week period, the DM+EX mice engaged in treadmill exercise (ZH-PT, Anhui Zhenghua Biological Instrument Co., Ltd. Huaibei, China) at a speed of 15 m/min for 20 min per session, 6 days per week.

### 2.3. Oral Glucose Tolerance Test (OGTT) and Insulin Tolerance Test (ITT)

Tail vein blood samples were collected to conduct both the OGTT and ITT. For the OGTT, the mice were fasted for 12–14 h before receiving an oral glucose dose of 2 g/kg. Blood glucose levels were measured at 0, 30, 60, 90, and 120 min using an Accu—Chek glucose meter (Roche, Roche Diabetes Care GmbH, Raunheim, Germany). For the ITT, the mice were administered an intraperitoneal insulin injection (1 U/kg) after a 4 h fast, with glucose measurements taken at the same time points. The area under the curve (AUC) was calculated to evaluate the results of both tests.

### 2.4. Serum Analysis

The activities of glycated serum protein (GSP) and tartrate-resistant acid phosphatase (TRAP) were measured using commercial assay kits (Nanjing Jiancheng Bioengineering Institute, Nanjing, China), following the manufacturer’s instructions. Serum levels of insulin, osteocalcin (OCN), carboxylated osteocalcin (cOC), and its undercarboxylated form (ucOC) were evaluated using ELISA kits (Shanghai Enzyme-linked Biotechnology Co., Ltd., Shanghai, China). A microplate reader (Bio-Rad, Hercules, CA, USA) was used to evaluate the optical density.

### 2.5. Morphological Evaluation

Pancreatic tissues were dehydrated through a graded ethanol series and embedded in paraffin. The embedded tissues were sectioned to a thickness of 8–10 μm using a microtome (Dako, Tokyo, Japan). After drying and dewaxing, the slices were stained with hematoxylin and eosin (H&E). Femoral samples were processed for histological analysis (H&E staining and TRAP staining) [29]. Images of stained sections were acquired with an inverted microscope (Olympus IX73, Tokyo, Japan). Osteoblasts and osteoclasts in femoral tissues were quantified using Image-Pro Plus 6.0 software.

### 2.6. Mechanical Properties

The mechanical characteristics of the femurs were evaluated using a universal testing machine (WDW-5, Jinan Tianhua Testing Equipment Co., Ltd., Jinan, China). Each bone was positioned across two supports spaced 10 mm apart, and a load was applied to the central region of the shaft. The load was applied at a constant speed of 2 mm/min until fracture occurred, and the load–displacement data were recorded. Maximum load, stiffness, elastic modulus, and energy absorption of femur were calculated from these data.

### 2.7. Microstructural Properties of Femur

Femoral microstructure was estimated using a micro-computed tomography (Micro-CT; Y. Cheetah; YXLON International GmbH, Hamburg, Germany) and dual-energy X-ray absorptiometry (DXA Insight Vet DXA, Osteosys, Seoul, Republic of Korea). Micro-CT images were rebuilt and analyzed with VG Studio MAX 3.0 software to define a femoral region of interest, allowing for quantification of trabecular number (Tb.N), trabecular thickness (Tb.Th), trabecular separation (Tb.Sp), and cortical bone thickness. For DXA measurements, mice were anesthetized with sodium pentobarbital (30 mg/kg) and placed supine on the scanner with hind limbs extended. The manufacturer’s software was then used to determine whole body and femoral bone mineral density (BMD), bone mineral content (BMC), and bone volume (BV).

### 2.8. Cell Experiments

#### 2.8.1. Cell Culture and Mechanical Stretch Treatment

As previously described [30], MC3T3-E1 cells (obtained from Xi’an Jiaotong University, Xi’an, China) were subjected to mechanical stretch intervention. The cells were cultured in α-MEM and, upon reaching 90% confluence, were trypsinized using 0.25% trypsin-EDTA and seeded onto a sheet (79 × 40 × 1.38 mm) at a density of 2 × 10^4^ cells cm^−2^. Once cells attached to the sheet, the culture medium was switched to a high-glucose formulation (α-MEM supplemented with 10% FBS, 1% penicillin–streptomycin, and 50 mmol L^−1^ glucose [31]). The cells were then divided into normal control (NC), high-glucose (HG), high-glucose medium containing 200 ng mL^−1^ insulin (HG+INS) [32], and high-glucose medium with mechanical treatment (HG+EX). For five consecutive days, the HG+EX group underwent cyclic mechanical stretch (2000 μstrain, 0.5 Hz, 60 min/day) using a four-point bending device (SXG4201, Chengdu Mirui Science and Technology Co. Ltd., Chengdu, China). After the intervention, the cells were harvested for further analysis.

#### 2.8.2. Construction and Transfection of the *Piezo1*-sgRNA Vector

To explore the role of Piezo1 in mediating the effects of exercise on osteoporosis and hyperglycemia in T1DM, a CRISPR-Cas9 strategy was employed to generate an MC3T3-E1 cell line with stable *Piezo1* knockout (*Piezo1*^−/−^). Using the enhanced CRISPR design tool (http://crispr.mit.edu (accessed on 1 March 2025)), we established single-guide RNAs (sgRNAs) targeting the proximal promoter region of the murine *Piezo1* gene. The oligonucleotides sequences for the selected sgRNA were as follows:

Sense: 5′-CACCGAGAGAGCATTGAAGCGTAAC-3′;

Antisense: 3′-CTCTCTCGTAACTTCGCATTGCAAA-5′.

The oligonucleotides were annealed and cloned into the PX459 vector. The recombinant plasmid was transfected into MC3T3-E1 cells using transfection reagent according to the manufacturer’s instructions. Finally, *Piezo1* knockout was verified by Sanger sequencing of the genomic DNA across the target site and by protein analysis to verify the absence of Piezo1. The verified *Piezo1*^−/−^ cells was cultured in α-MEM supplemented with 10% fetal bovine serum and 1% penicillin/streptomycin (Servicebio, Wuhan, China). The cells were maintained a humidified incubator with 5% CO_2_ at 37 °C. For subsequent experiments, *Piezo1*^−/−^ cells were allocated into the following three experimental groups:

NC + *Piezo1*^−/−^: MC3T3-E1 cells grown in culture medium; HG + *Piezo1*^−/−^: MC3T3-E1 cells grown in medium with high level of glucose; and HG + EX + *Piezo1*^−/−^: MC3T3-E1 cells grown in medium with high level of glucose and subjected to exercise treatment.

#### 2.8.3. Cell Proliferation

Following the protocol provided with the CCK-8 kit (NCM Biotech, Suzhou, China) cellular proliferation was assessed. Absorbance was estimated using a microplate reader (Bio-Rad, Hercules, CA, USA) at 450 nm. Each sample was performed in triplicate to ensure the accuracy and reliability of the data.

#### 2.8.4. Cell Differentiation

Cell differentiation of cells was evaluated using Alizarin Red S staining (Solarbio, Beijing, China) and alkaline phosphatase (ALP) activity. Alizarin Red S staining was performed according to the procedure of previous report [29], allowing visualization of calcium mineralized nodules under an inverted light microscope (Olympus IX73, Tokyo, Japan). ALP activity was quantified using a commercial assay kit (Nanjing Jiancheng Bioengineering Institute, Nanjing, China) according to the manufacturer’s directions. Absorbance was measured at 520 nm with a microplate reader (Bio-Rad, Hercules, CA, USA).

#### 2.8.5. Fluorescence Staining

Following the 5-day treatment, cells were rinsed with PBS and fixed with 4% PFA for 15 min. Cells were then treated with 0.5% Triton X-100 solution for 10 min at room temperature. After another PBS wash, F-actin was stained by incubating the cells with FITC-phalloidin solution for 20 min at room temperature. Nuclei were subsequently stained with DAPI solution (Servicebio, Wuhan, China) for 15 min. Fluorescence images were captured using an inverted microscope (Olympus IX73, Tokyo, Japan).

### 2.9. Protein Analysis

Protein extraction and analysis from bone tissues and cells were carried out as previously described [33]. Immunoreactive bands were visualized using an ECL-plus reagent (BC1101, ZHHC Biopharmaceutical Technology, Xi’an, China) and imaged with an Azure Biosystems C300 imaging system (Azure Biosystems, Dublin, CA, USA). Signal intensities were quantified using Image Lab software (version 6.1). Details of the antibody used are provided in the Supplementary Materials Table S6.

### 2.10. Statistical Analysis

Statistical analyses were carried out using SPSS version 25.0 (IBM, Armonk, NY, USA) and GraphPad Prism 8.0 software (GraphPad, Inc., La Jolla, CA, USA). Data were initially assessed for normality and homogeneity of variance, followed by parametric post hoc analysis to confirm a priori power calculations. To evaluate differences between groups, a one-way analysis of variance was performed, followed by Tukey’s post hoc test for pairwise comparisons. Dunnett’s test was used to assess the statistical significance of cell viability data. *p*-Values < 0.05 were considered statistically significant.

## 3. Results

### 3.1. Physiological and Metabolic Profiles of Mice

**Figure 1:**
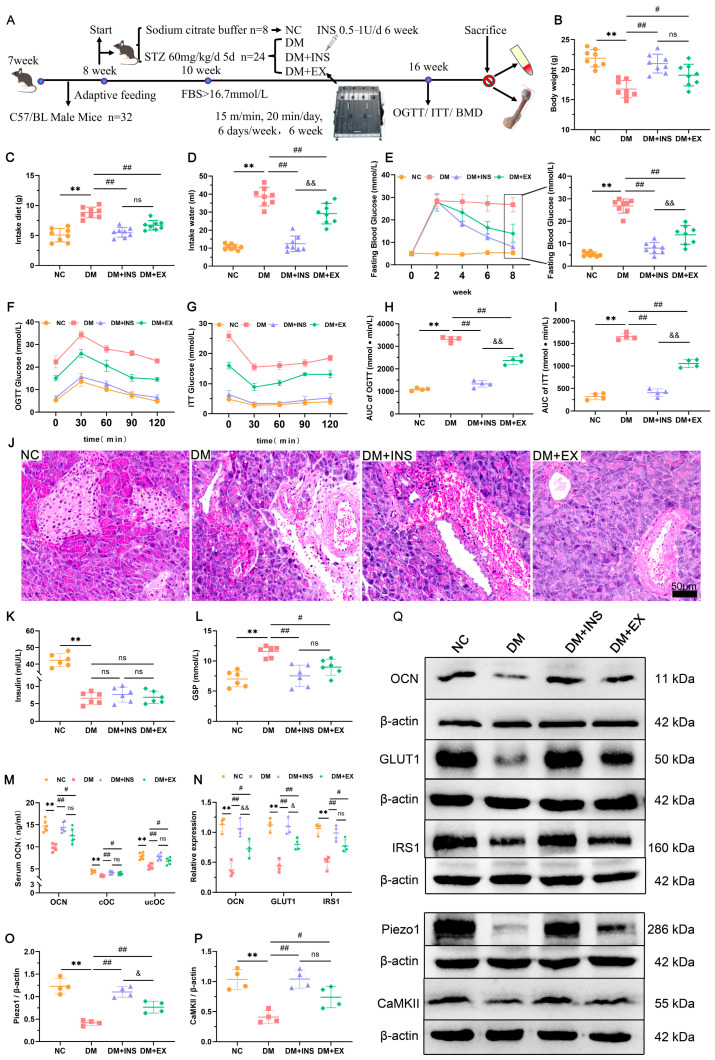
Physiological and biochemical properties of mice: (**A**) schematic diagram of experimental protocol; (**B**) body weight of mice; (**C**) intake diet; (**D**) intake water; (**E**) fasting blood glucose (*n* = 8); (**F**) OGTT glucose; (**G**) ITT glucose; (**H**) area under the curve of OGTT; (**I**) area under the curve of ITT (*n* = 4); (**J**) pancreas tissues were observed using H&E staining, scale bars, 50 μm; (**K**) serum insulin levels; (**L**) serum GSP levels; (**M**) serum OCN, cOC, ucOC levels (*n* = 6); (**N**) Expression level of OCN, GLUT1, and IRS1 protein; (**O**) Expression level of Piezo1 protein; (**P**) Expression level of CaMKII protein (*n* = 4); (**Q**) Protein bands of signaling molecules. Individual data points are shown as the mean ± SD. NC vs. DM: ** *p* < 0.01; DM+INS vs. DM, DM+EX vs. DM: ^#^ *p* < 0.05, ^##^ *p* < 0.01; DM+INS vs. DM+EX: ^&^ *p* < 0.05, ^&&^ *p* < 0.01; ns, no significance. The original Western blot images are summarized in Table S1.

Following one week of acclimation, T1DM was induced in mice, after which they underwent a 6-week exercise intervention (Figure 1A). Compared with the NC, DM mice exhibited reduced body weight, alongside polyphagia and polydipsia (Figure 1B–D). STZ administration resulted in sustained and significant hyperglycemia in all diabetic mice, with their FBG levels remaining consistently elevated relative to the NC group throughout the subsequent 6-week period (Figure 1E). Moreover, the DM group exhibited markedly compromised glucose tolerance and insulin resistance, as evidenced by higher AUC values in both the OGTT and ITT (Figure 1F–I).

After six weeks treatment, exercise partially alleviated the metabolic disturbances observed in the diabetic mice. Specifically, DM+EX mice exhibited significant reductions in diet intake, water intake, FBG levels, and the AUC values for both the OGTT and ITT (Figure 1C–I). Additionally, body weight in the DM+EX group was markedly increased compared with the DM group (Figure 1B). Although exercise normalized body weight to levels comparable with the DM+INS group, its ability to fully normalize overall metabolic homeostasis was less effective than that of insulin. Compared with the DM+INS group, the DM+EX group continued to exhibit higher food and water intake, FBG levels, and AUC values in both the OGTT and ITT.

Histological examination revealed morphological abnormalities in the pancreatic islets of the DM group, including irregular contours and vacuolization, contrasting with the well-defined islets observed in the NC group (Figure 1J). Compared with the NC group, the DM group showed significantly reduced serum levels of insulin, OCN, cOC, and ucOC, alongside elevated GSP levels (Figure 1K–M). Notably, exercise intervention failed to reverse the pancreatic islet pathology, and the associated increase in insulin levels was not statistically significant. Exercise, however, significantly increased serum levels of OCN, cOC, and ucOC, while reducing GSP levels (Figure 1K–M). Aligning with the serum observations, the DM group exhibited markedly lower protein expression of OCN, GLUT1, and insulin receptor substrate 1 (IRS1) compared with the NC group (Figure 1N,Q). Exercise significantly upregulated the expression of OCN, GLUT1, and IRS1 of DM mice. Nevertheless, despite these exercise-induced elevations, their expression remained below the levels observed in the DM+INS group. Similarly, compared with the NC group, the DM group showed significantly reduced protein signal intensities for Piezo1 and CaMKII (Figure 1O–Q), whereas exercise intervention significantly upregulated both proteins compared with the DM group.

### 3.2. Structural Properties of Femur

**Figure 2:**
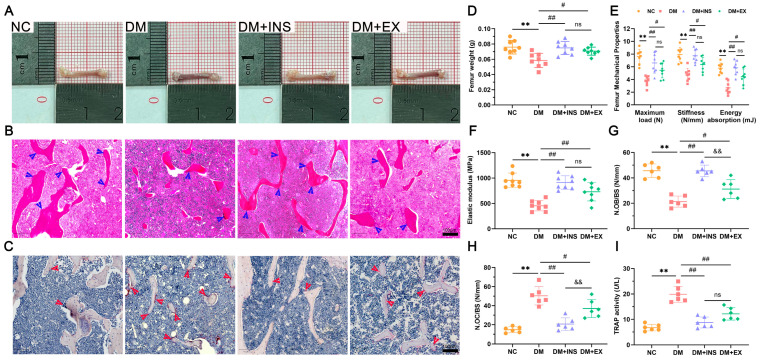
Morphological and Mechanical characteristics of femur: (**A**) photographs of femur; (**B**) number of osteoblasts (blue arrows) were observed using H&E staining; (**C**) number of osteoclasts (red arrows) were observed using TRAP staining, scale bar, 100 μm; (**D**) femur weight; (**E**) maximum load, stiffness, and energy absorption; (**F**) elastic modulus (*n* = 8); (**G**) N.OB/BS; (**H**) N.OC/BS; (**I**) serum TRAP activity (*n* = 6). Individual data points are shown as the mean ± SD. NC vs. DM: ** *p* < 0.01; DM+INS vs. DM, DM+EX vs. DM: ^#^ *p* <0.05, ^##^ *p* < 0.01; DM+INS vs. DM+EX: ^&&^ *p* < 0.01; ns: no significance. The original figures of TRAP are provided in the Supplementary Materials Table S7.

Macroscopically, the femurs of DM+EX mice were longer and heavier than those of DM mice (Figure 2A,D). The mechanical properties—including maximum load, elastic modulus, stiffness, and energy absorption—of the femur in T1DM mice were improved by exercise (Figure 2E,F). H&E and TRAP staining were carried out to assess femoral bone microarchitecture (Figure 2B,C). Quantitative analyses further revealed that, compared with the NC group, the DM group exhibited a significantly lower number of osteoblasts per bone surface (N.OB/BS) (Figure 2G). Conversely, the DM group showed a higher number of osteoclasts per bone surface (N.OC/BS) and significantly elevated TRAP activity (Figure 2H,I). In summary, exercise increased osteoblast number and significantly suppressed osteoclast activity and TRAP activation.

**Figure 3:**
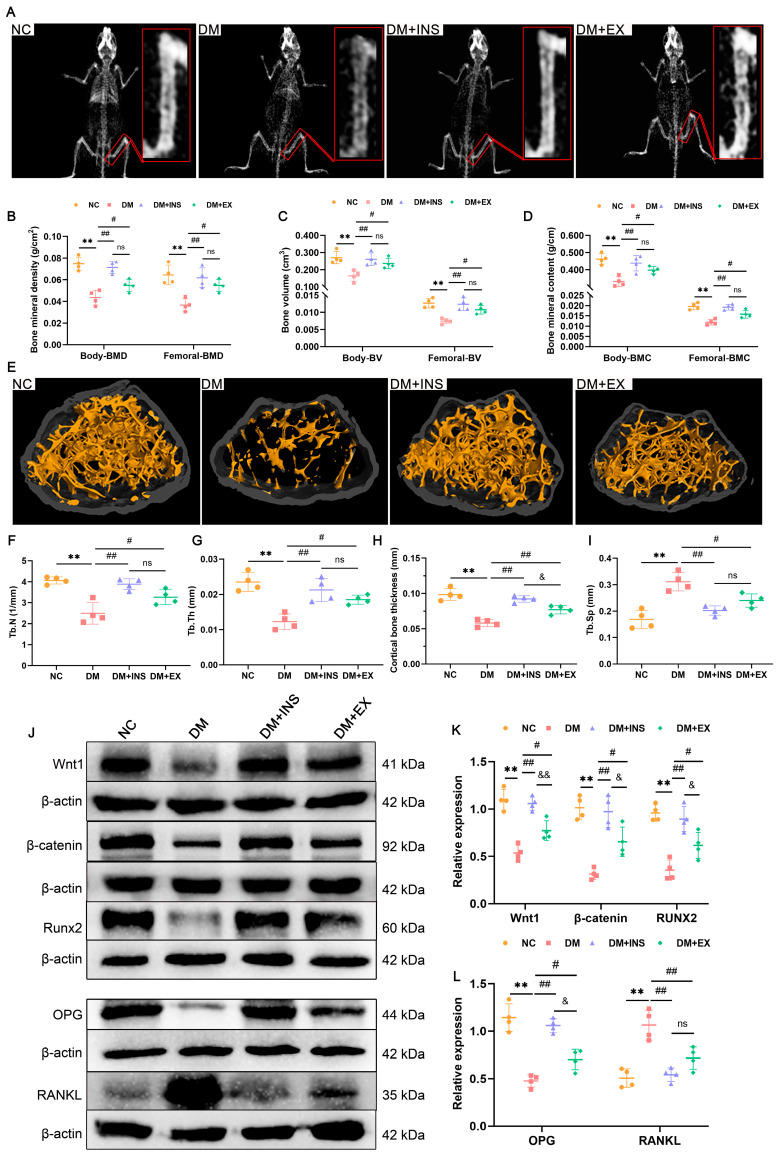
Microstructure properties and protein expression of femur: (**A**) representative 2D DXA images of mice; (**B**) BMD; (**C**) BV; (**D**) BMC; (**E**) representative 3D micro-CT images of femur; (**F**) Tb.N: (**G**) Tb.Th; (**H**) cortical bone thickness; (**I**) Tp.Sp; (**J**) protein bands of osteogenic and osteoclast molecules; (**K**) protein expression of osteogenic molecules; (**L**) protein expression of osteoclast molecules. (*n* = 4). Individual data points are shown as the mean ± SD. NC vs. DM: ** *p* < 0.01; DM+INS vs. DM, DM+EX vs. DM: ^#^ *p* < 0.05, ^##^ *p* < 0.01; DM+INS vs. DM+EX: ^&^ *p* < 0.05, ^&&^ *p* < 0.01; ns, no significance. The original Western blot images are summarized in Table S2.

Correspondingly, the NC group exhibited an intact, well-organized trabecular bone structure with narrow trabecular spacing in the distal femur. Femurs of the NC group displayed stable, dense, and undistorted structure, while those from the DM mice were thinner, less dense, and showed signs of distortion (Figure 3A). Three-dimensional reconstruction further confirmed the microstructural deterioration of the femur in the DM group, which displayed decreased trabecular bone number, trabecular bone thickness, and cortical bone thickness, along with increased trabecular separation and disorganized architecture compared with the NC group (Figure 3E–I). The DM group exhibited severe structural damage, characterized by reduced trabecular number and increased spacing. Compared with the NC group, the DM mice showed significantly lower whole-body and femoral BV, BMD, and BMC, further highlighting these structural alterations (Figure 3B–D). Consistent with these morphological alterations, protein expression of Wnt1, β-catenin, Runx2, and OPG in bone tissue were significantly lower in the DM group compared with the NC group, while the expression of RANKL was significantly higher (Figure 3J–L). Following exercise intervention, the DM+EX group demonstrated denser and more orderly trabecular bone architecture, alongside significant improvements in multiple bone parameters and protein expression relative to the DM group. Moreover, exercise promoted an osteogenic environment, as evidenced by the upregulation of key osteogenic markers—Wnt1, β-catenin, and Runx2—and a significant increase in the OPG/RANKL ratio in the DM+EX group compared with the DM group.

### 3.3. Mechanical Stretch for Cells

**Figure 4:**
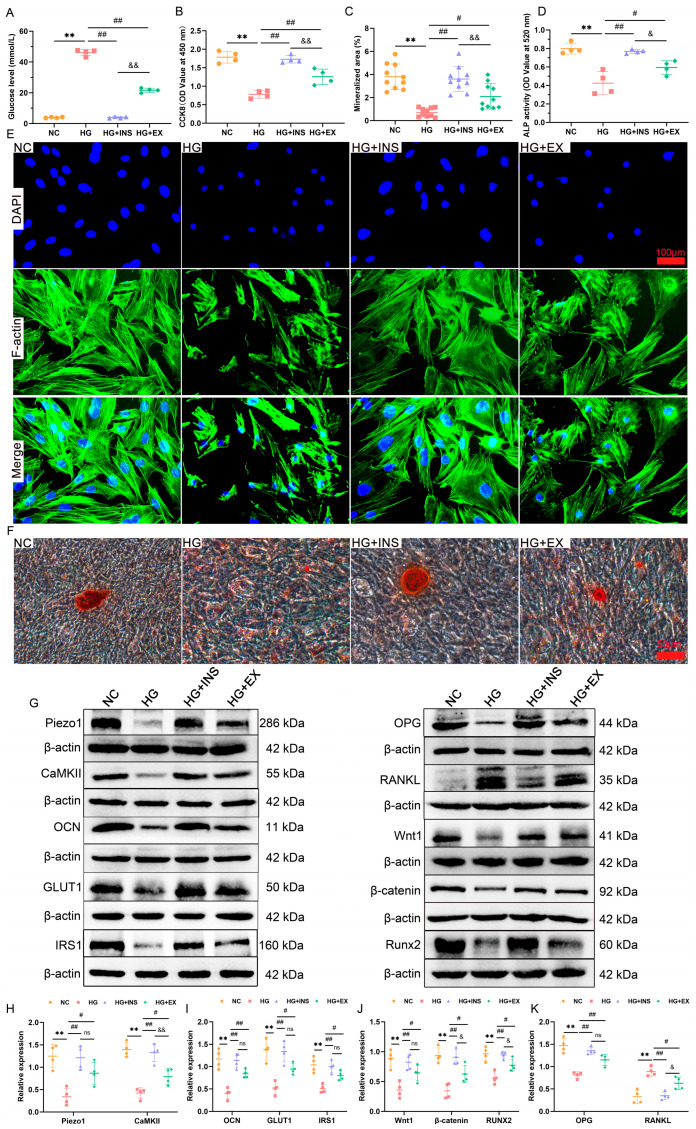
Mechanical stretch on proliferation, differentiation and protein expression of MC3T3-E1 cell: (**A**) glucose concentration in cell culture medium; (**B**) absorbance value (OD) of cell viability (*n* = 4); (**C**) quantitative evaluation of alizarin red S deposition (*n* = 10); (**D**) absorbance value (OD) of ALP activity; (**E**) blue fluorescence staining: nuclei with DAPI, green fluorescence staining: F-actin, scale bar, 100 μm; (**F**) representative images of alizarin red S staining assay, scale bar, 50 μm; (**G**) representative images of WB bands; (**H**) expression level of Piezo1 and CaMKII protein; (**I**) expression level of OCN, GLUT1, and IRS1 protein; (**J**) expression level of Wnt1, β-catenin, and Runx2 protein; (**K**) expression level of OPG and RANKL protein (*n* = 4). Individual data points are shown as the mean ± SD. NC vs. HG: ** *p* < 0.01; HG+INS vs. HG, HG+EX vs. HG: ^#^ *p* <0.05, ^##^ *p* < 0.01; HG+INS vs. HG+EX: ^&^ *p* < 0.05, ^&&^ *p* < 0.01; ns, no significance. The original Western blot images are summarized in Table S3.

In cellular experiments, mechanical stretch was applied to MC3T3-E1 cells to mimic the mechanical stimulation induced by exercise. Compared with the NC group, the HG group exhibited a significant decrease in cell proliferation (Figure 4B) and osteogenic differentiation capacity. This impairment in osteogenic differentiation was evidenced by reduced mineralization (Figure 4C,F), decreased ALP activity (Figure 4D), and disorganized F-actin cytoskeletal structure (Figure 4E). Conversely, the HG group showed a significantly higher glucose concentration in the culture supernatant than the NC group (Figure 4A). Mechanical stretch (HG+EX group) significantly ameliorated these high glucose-induced impairments. Specifically, stretch enhanced cell proliferation and osteogenic differentiation, as indicated by increased mineralization and ALP activity. Furthermore, it improved cytoskeletal morphology and reduced extracellular glucose concentration compared with the non-stretched HG group.

Consistent with the in vivo findings, HG conditions markedly reduced the protein expression of Piezo1, CaMKII, Wnt1, β-catenin, Runx2, OPG, OCN, GLUT1, and IRS1 in MC3T3-E1 cells compared with the NC group (Figure 4G–K). Conversely, RANKL expression was upregulated under HG conditions. Mechanical stretch intervention effectively counteracted these alterations, significantly increasing the expression of the downregulated proteins while suppressing RANKL expression compared with the HG group. Nevertheless, the stimulatory effect of mechanical stretch on most of these proteins was still less pronounced than the effect of insulin administration.

**Figure 5:**
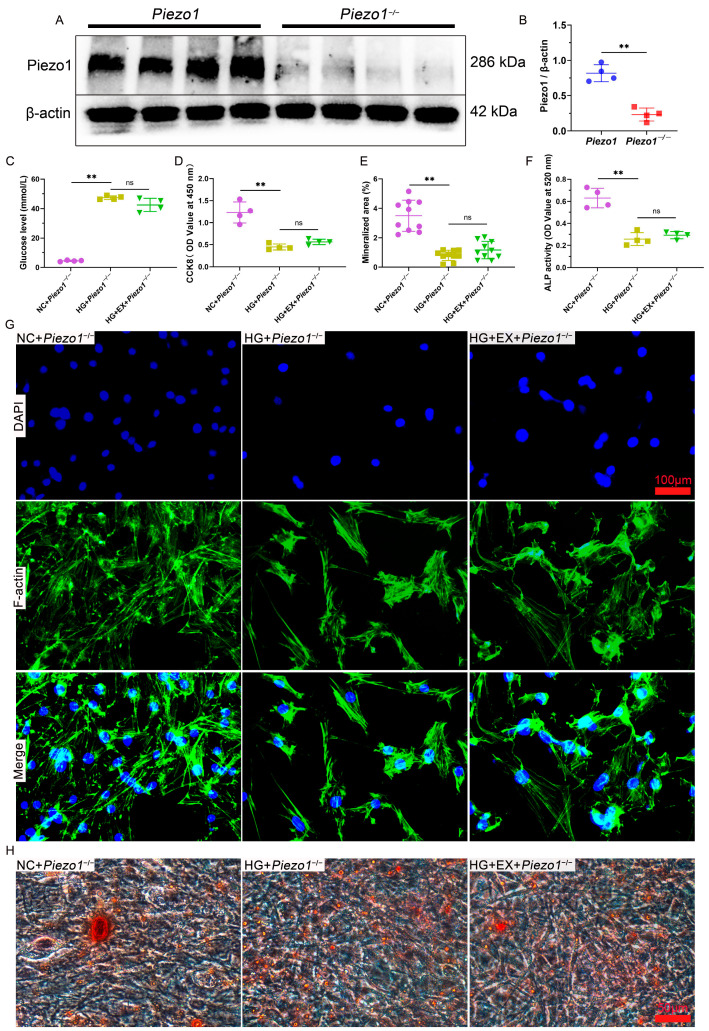
Mechanical stretch on proliferation and differentiation of *Piezo1*^−/−^ cells: (**A**) Western blot bands of Piezo1; (**B**) expression level of Piezo1 protein; (**C**) glucose concentration in cell culture medium; (**D**) absorbance value (OD) of cell viability (*n* = 4); (**E**) quantitative evaluation of alizarin red S deposition (*n* = 10); (**F**) The absorbance value (OD) of ALP activity (*n* = 4); (**G**) blue fluorescence staining: nuclei with DAPI, green fluorescence staining: F-actin, scale bar,100 μm; (**H**) representative images of alizarin red S staining assay, scale bar, 50 μm. Individual data points are shown as the mean ± SD. *Piezo1* vs. *Piezo1*^−/−^, NC+ *Piezo1*^−/−^ vs. HG+ *Piezo1*^−/−^: ** *p* < 0.01; ns, no significance. The original Western blot images are summarized in Table S4.

**Figure 6:**
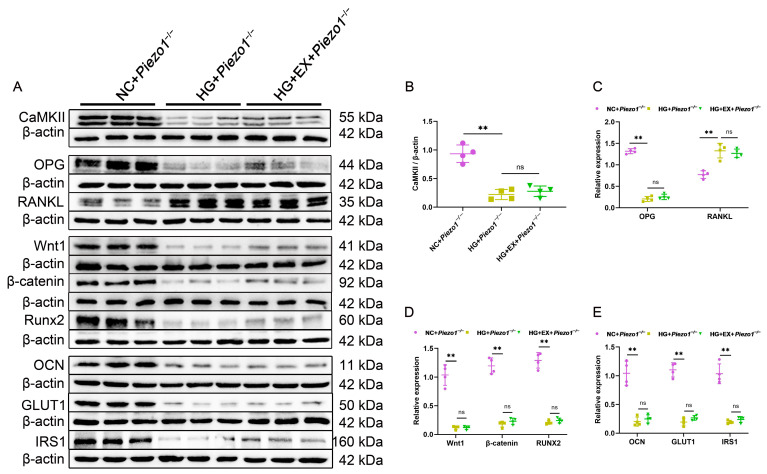
Mechanical stretch on protein expression of *Piezo1*^−/−^ cells: (**A**) representative images of western blot bands; (**B**) expression level of CaMKII protein; (**C**) expression level of OPG and RANKL protein; (**D**) expression level of Wnt1, β-catenin, and Runx2 protein; (**E**) expression of OCN, GLUT1, and IRS1 protein (*n* = 4). Individual data points are shown as the mean ± SD. NC+ *Piezo1*^−/−^ vs. HG+ *Piezo1*^−/−^: ** *p* < 0.01; ns, no significance. The original Western blot images are summarized in Table S5.

To evaluate whether Piezo1 mediates the beneficial effects of exercise, we developed a MC3T3-E1 cell model with *Piezo1*-knockout. Western blot analysis confirmed the successful *Piezo1*-knockout (*Piezo1*^−/−^), as evidenced by the near absence of the Piezo1 protein band (Figure 5A,B). In cells cultured under HG conditions, mechanical stretch applied to the HG+EX+*Piezo1*^−/−^ group (cells cultured in high glucose medium and subjected to mechanical stretch) induced only slightly increases in cell proliferation, F-actin intensity, ALP activity, and calcified nodule formation relative to the HG+*Piezo*1^−/−^ group (Figure 5D–H). Correspondingly, stretch resulted in only a slight decrease in glucose concentration in the extracellular matrix (Figure 5C). Western blot further revealed that, compared with the HG+*Piezo1*^−/−^ group, mechanical stretch in the HG+EX+*Piezo1*^−/−^ group resulted in only minimal changes in CaMKII, OPG, Wnt1, β-catenin, Runx2, OCN, GLUT1, and IRS1, along with a modest reduce in RANKL expression (Figure 6).

## 4. Discussion

Diabetic osteoporosis, a severe complication of T1DM, significantly increases fracture risk due to prolonged disease duration and poor glycemic control [34]. Although pharmacological treatments are available, their side effects remain a concern. Exercise provides a multifaceted alternative by improving metabolic and immunomodulatory outcomes [35]. To elucidate the mechanism by which exercise concurrently alleviates T1DM-induced osteoporosis and hyperglycemia, we established a stable mouse model. STZ-injected mice exhibited sustained hyperglycemia (FBG ≥16.7 mmol L^−1^), classic diabetic symptoms (weight loss, polyuria, and polydipsia), and irregular pancreatic islet morphology—features that were not altered by exercise—confirming successful model establishment [36].

A 6-week exercise intervention induced significant metabolic and skeletal improvements in T1DM mice. Exercise partially alleviated hyperglycemia and classic diabetic symptoms while improving glucose tolerance and insulin sensitivity (reduced AUC in OGTT and ITT). Structurally, exercise promoted denser and more orderly trabecular architecture and restored femoral bone microarchitecture, leading to improved mechanical properties of the femur. Collectively, these results show that exercise alleviates T1DM-induced osteoporosis by improving both bone morphology and mechanical strength.

Mechanical stimulation regulates bone remodeling, a process that maintains a balance between osteoblast-mediated bone formation and osteoclast-mediated bone resorption. The Wnt/β-catenin signaling pathway functions a critical role in increasing osteoblast proliferation, and its dysregulation contributes to the pathogenesis of osteoporosis [37,38]. Our results demonstrate that exercise upregulates key components of this pathway—Wnt1, β-catenin, and the osteogenic transcription factor Runx2—thereby promoting bone formation. Conversely, bone resorption is governed by the OPG/RANKL axis, wherein a higher OPG/RANKL ratio inhibits osteoclastogenesis [39]. At the protein level, exercise consistently increased the expression of Wnt1, β-catenin, and Runx2, while elevating the OPG/RANKL ratio. This dual mechanism—activating the Wnt/β-catenin/Runx2 axis to promote bone formation and modulating the OPG/RANKL axis to suppress bone resorption—underlies the osteoprotective effect of exercise in T1DM.

Glucose uptake is essential for cellular survival and function. In diabetes, hyperglycemia coincides with impaired glucose uptake in pancreatic β-cells. IRS1, a key adaptor protein in insulin signaling, regulates insulin-mediated glucose uptake and β-cell function [40]. While increased IRS1 enhances insulin-stimulated glucose clearance in peripheral tissues, it cannot stimulate insulin secretion from damaged β-cells in T1DM. Therefore, the reduction in blood glucose observed in exercised T1DM mice is likely mediated by insulin-independent pathways. OCN, an osteoblast-derived endocrine hormone, plays a role in energy metabolism [41]. Its circulating levels positively correlate with glycemic control [42,43], and OCN-deficient mice exhibit hyperglycemia [43]. In this study, a 6-week exercise intervention effectively upregulated both OCN and its putative downstream effector, GLUT1, in T1DM mice. These findings demonstrate that the exercise-induced increase in serum OCN is associated with improved glycemic control, supporting the concept of the skeleton as an endocrine organ that regulates systemic glucose homeostasis via OCN. Collectively, exercise enhances glucose transport capacity, at least in part, by upregulating the OCN–GLUT1 axis in T1DM mice.

The skeletal system, the body’s primary mechanical structural framework, is constantly subjected to mechanical stimulation. It maintains homeostasis through mechanotransduction, the process by which it adapts its structure in response to these stimuli [44]. This process is essential for bone physiology, including growth and remodeling, and its dysregulation contributes to skeletal disorders. The principal mediator of mechanotransduction is Piezo1, a mechanosensitive ion channel that converts mechanical signals such as shear stress into biochemical responses [45]. Piezo1 is crucial for mediating the skeletal anabolic effects of mechanical loading. In animal models, its specific ablation in osteoblasts induces an osteoporotic phenotype [46], highlighting its indispensable role in bone metabolism.

We hypothesized that Piezo1, a key mechanosensitive ion channel on bone cells, transduces exercise-induced mechanical signals to activate downstream anabolic pathways. Accordingly, we investigated the role of Piezo1 in this exercise-induced adaptive response. Our findings demonstrate that Piezo1-mediated mechanotransduction is essential for exercise-induced bone formation. Piezo1 transduces mechanical signals primarily through Ca^2+^ influx, which subsequently activates downstream signaling pathways [47]. Specifically, Ca2+ influx through activated Piezo1 implicates CaMKII as a key downstream mediator [28], and the Piezo1-CaMKII axis is well established as a critical regulator in mechanical stress-related bone biology [48]. In our study, exercise intervention significantly upregulated CaMKII protein expression, a pattern consistent with that of Piezo1.

In Vitro, mechanical stretch under high-glucose conditions significantly enhanced osteoblast proliferation, differentiation, and mineralization, while reducing extracellular glucose levels. However, these therapeutic benefits were almost entirely abrogated in *Piezo1*^−/−^ cells. Compared with the non-stretched HG+*Piezo1*^−/−^ controls, mechanical stretch conferred only marginal improvements in both osteogenic capacity and glucose-lowering efficacy. In summary, these results exhibit that the positive impacts of exercise on bone remolding and glucose metabolism substantially rely on Piezo1-mediated mechanotransduction. While exercise provides therapeutic benefits, its effects on ameliorating osteoporosis and hyperglycemia associated with T1DM are not as pronounced as those achieved with insulin treatment.

Beyond its direct mechanical effects on osteoclasts/osteoblasts, exercise-induced bone regulation may involve complex indirect pathways, particularly through the nervous system [49]. Exercise is known to modulate autonomic outflow and the neuroendocrine axis, such as the sympathetic nervous system and the IGF-1 signaling pathway, both critical for bone homeostasis. While our study primarily focused on Piezo1 in osteoblasts, it is noteworthy that Piezo1 is also expressed in sensory neurons innervating the bone matrix. This raises the possibility that exercise-induced mechanical loading may activate neuronal Piezo1, triggering neurogenic signals that synergize with direct mechanical stimulation to promote bone formation. Furthermore, the crosstalk between the skeletal and nervous systems may be bidirectional. As a functional endocrine organ, the skeleton secretes hormones like osteocalcin, which regulates central glucose metabolism and energy expenditure. Our findings that exercise activates Piezo1 in osteoblasts suggest a potential upstream mechanism: Piezo1-mediated mechanotransduction might facilitate the synthesis or release of bioactive osteocalcin, thereby indirectly modulating systemic metabolism via the neuroendocrine route.

Although the present study focused on Piezo1 in osteoblasts, this mechanosensitive ion channel may also be expressed in sensory neurons innervating the bone matrix. However, this study did not experimentally dissect the potential contribution of Piezo1 expressed in sensory neurons innervating bone. Additionally, the mechanical loading paradigm used in cell experiments may not fully capture the complexity of physiological exercise. Future research should aim to integrate multi-system approaches to confirm whether Piezo1 activation in bone can, indeed, influence systemic metabolic regulation via neuronal circuits. Despite these constraints, this study offers a foundational understanding of Piezo1-mediated mechanotransduction and opens new interdisciplinary perspectives linking skeletal biology to neuroendocrinology.

## 5. Conclusions

This study investigated exercise training as a therapy for T1DM-induced osteoporosis and hyperglycemia. The results show that a 6-week exercise regimen provided a strong dual benefit, simultaneously alleviating osteoporosis and reducing hyperglycemia in T1DM mice, as evidenced by improved femoral biomechanics, morphology, and microstructure. Although effective, exercise was generally less efficacious than insulin therapy. Mechanistically, all experiments revealed that the osteogenic and glucose-lowering effects of exercise or mechanical stretch are primarily mediated by the Piezo1 signaling pathway, as its genetic knockout significantly diminished these benefits. This work establishes Piezo1-mediated mechanotransduction as the critical link connecting mechanical stimuli to concurrent improvement of the skeletal–metabolic axis in T1DM. Our findings suggest new therapeutic strategies of T1DM management that leverage Piezo1-mediated mechanotransduction.

## Data Availability

All data will be made available upon reasonable request.

## Supplementary Materials

The following supporting information can be downloaded at: https://www.mdpi.com/article/10.3390/biology15110819/s1, Table S1: The original Western blot images for Figure 1; Table S2: The original Western blot images for Figure 3; Table S3: The original Western blot images for Figure 4; Table S4: The original Western blot images for Figure 5; Table S5: The original Western blot images for Figure 6; Table S6: The antibody information. Table S7: The original figures of TRAP.

## References

[1] Katsarou A., Gudbjornsdottir S., Rawshani A., Dabelea D., Bonifacio E., Anderson B.J., Jacobsen L.M., Schatz D.A., Lernmark A. (2017). Type 1 diabetes mellitus. Nat. Rev. Endocrinol..

[2] Pan Q., Chen H., Fei S., Zhao P., Deng M., Xiao F., Guo L. (2023). Medications and medical expenditures for diabetic patients with osteoporosis in Beijing, China: A retrospective study. Diabetes Res. Clin. Pract..

[3] Pujia A., Gazzaruso C., Montalcini T. (2017). An update on the potential role of C-peptide in diabetes and osteoporosis. Endocrine.

[4] Shiel E.V., Hemingway S., Burton K., King N. (2023). Self-management of type 1 diabetes in young adults: Is it impeded by aspects of everyday life? A scoping review. Diabetes Metab. Syndr..

[5] DiMeglio L.A., Evans-Molina C., Oram R.A. (2018). Type 1 diabetes. Lancet.

[6] Khosla S., Hofbauer L.C. (2017). Osteoporosis treatment: Recent developments and ongoing challenges. Lancet Diabetes Endocrinol..

[7] James A.W., LaChaud G., Shen J., Asatrian G., Nguyen V., Zhang X., Ting K., Soo C. (2016). A review of the clinical side effects of bone morphogenetic protein-2. Tissue Eng. Part B Rev..

[8] Barry M., Pearce H., Cross L., Tatullo M., Gaharwar A.K. (2016). Advances in nanotechnology for the treatment of osteoporosis. Curr. Osteoporos. Rep..

[9] Zheng Z., Yu C.Y., Wei H. (2021). Injectable hydrogels as three-dimensional network reservoirs for osteoporosis treatment. Tissue Eng. Part B Rev..

[10] Macias I., Alcorta-Sevillano N., Rodriguez C.I., Infante A. (2020). Osteoporosis and the potential of cell-based therapeutic strategies. Int. J. Mol. Sci..

[11] Aibar-Almazan A., Voltes-Martinez A., Castellote-Caballero Y., Afanador-Restrepo D.F., Carcelen-Fraile M.D.C., Lopez-Ruiz E. (2022). Current status of the diagnosis and management of osteoporosis. Int. J. Mol. Sci..

[12] Fuchs S., Ernst A.U., Wang L.H., Shariati K., Wang X., Liu Q., Ma M. (2021). Hydrogels in emerging technologies for type 1 diabetes. Chem. Rev..

[13] Kioulaphides S., Garcia A.J. (2024). Encapsulation and immune protection for type 1 diabetes cell therapy. Adv. Drug Deliv. Rev..

[14] Morris A. (2018). Closed-loop insulin delivery has wide-ranging benefits. Nat. Rev. Endocrinol..

[15] White A.M., Shamul J.G., Xu J., Stewart S., Bromberg J.S., He X. (2020). Engineering strategies to improve islet transplantation for type 1 diabetes therapy. ACS Biomater. Sci. Eng..

[16] Shapiro A.M., Pokrywczynska M., Ricordi C. (2017). Clinical pancreatic islet transplantation. Nat. Rev. Endocrinol..

[17] Zhao Y., Jiang Z., Zhao T., Ye M., Hu C., Yin Z., Li H., Zhang Y., Diao Y., Li Y. (2012). Reversal of type 1 diabetes via islet beta cell regeneration following immune modulation by cord blood-derived multipotent stem cells. BMC Med..

[18] Park J., Wu Y., Li Q., Choi J., Ju H., Cai Y., Lee J., Oh Y.K. (2023). Nanomaterials for antigen-specific immune tolerance therapy. Drug Deliv. Transl. Res..

[19] Kwiatkowski A.J., Stewart J.M., Cho J.J., Avram D., Keselowsky B.G. (2020). Nano and microparticle emerging strategies for treatment of autoimmune diseases: Multiple sclerosis and type 1 diabetes. Adv. Healthc. Mater..

[20] Rasool N., Srivastava R., Singh Y. (2022). Cationized silica ceria nanocomposites to target biofilms in chronic wounds. Biomater. Adv..

[21] Aghebati-Maleki L., Dolati S., Zandi R., Fotouhi A., Ahmadi M., Aghebati A., Nouri M., Shakouri S.K., Yousefi M. (2019). Prospect of mesenchymal stem cells in therapy of osteoporosis: A review. J. Cell Physiol..

[22] Colberg S.R., Laan R., Dassau E., Kerr D. (2015). Physical activity and type 1 diabetes: Time for a rewire?. J. Diabetes Sci. Technol..

[23] Martnez-Ramonde T., Alonso N., Cordido F., Cervelló E., Cañizares A., Martínez-Peinado P., Sempere J.M., Roche E. (2014). Importance of exercise in the control of metabolic and inflammatory parameters at the moment of onset in type 1 diabetic subjects. Exp. Clin. Endocrinol. Diabetes.

[24] Chahal P.K., Bains K., Kaur H. (2023). Protective effect of indian herbs and physical exercise on osteoporosis: A review. Food Rev. Int..

[25] Choi S.B., Jang J.S., Park S.M. (2005). Estrogen and exercise may enhance β-cell function and mass via insulin receptor substrate 2 induction in ovariectomized diabetic rats. Endocrinology.

[26] Codella R., Terruzzi I., Luzi L. (2017). Why should people with type 1 diabetes exercise regularly?. Acta Diabetol..

[27] Qin L., He T., Chen S., Yang D., Yi W., Cao H., Xiao G. (2021). Roles of mechanosensitive channel Piezo1/2 proteins in skeleton and other tissues. Bone Res..

[28] Saotome K., Murthy S.E., Kefauver J.M., Whitwam T., Patapoutian A., Ward A.B. (2018). Structure of the mechanically activated ion channel Piezo1. Nature.

[29] Cui Y., Lv B., Li Z., Ma C., Gui Z., Geng Y., Liu G., Sang L., Xu C., Min Q. (2024). Bone-targeted biomimetic nanogels re-establish osteoblast/osteoclast balance to treat postmenopausal osteoporosis. Small.

[30] Zeng Q.C., Wang Y., Gao J., Yan Z.X., Li Z.H., Zou X.Q., Li Y.N., Wang J.H., Guo Y. (2019). miR-29b-3p regulated osteoblast differentiation via regulating IGF-1 secretion of mechanically stimulated osteocytes. Cell Mol. Biol. Lett..

[31] Hu S.J., Chen G.C., Wang F.Y., Fang Y.Q., Wang S.Q., Song Z.L., Zhao Z.H., Zhang Q.L., Meng X.Y., Zhang Q.Y. (2025). Network pharmacology analysis uncovers the mechanism of Shudihuang-Shanzhuyu herb pair in prevention and treatment of diabetic osteoporosis via PI3K/AKT pathway. J. Ethnopharmacol..

[32] Zhang N., Jiang H., Bai Y., Lu X.M., Feng M., Guo Y., Zhang S., Luo Q., Wu H., Wang L. (2019). The molecular mechanism study of insulin on proliferation and differentiation of osteoblasts under high glucose conditions. Cell Biochem. Funct..

[33] Xie Q., Du X., Liang J., Shen Y., Ling Y., Huang Z., Ke Z., Li T., Song B., Wu T. (2025). FABP4 inhibition suppresses bone resorption and protects against postmenopausal osteoporosis in ovariectomized mice. Nat. Commun..

[34] Weber D.R., Haynes K., Leonard M.B., Willi S.M., Denburg M.R. (2015). Type 1 diabetes is associated with an increased risk of fracture across the life span: A population-based cohort study using the health improvement network (THIN). Diabetes Care.

[35] Miller R.G., Mahajan H.D., Costacou T., Sekikawa A., Anderson S.J., Orchard T.J. (2016). A contemporary estimate of total mortality and cardiovascular disease risk in young adults with type 1 diabetes: The pittsburgh epidemiology of diabetes complications study. Diabetes Care.

[36] Li C.H., Gao Q.Y., Jiang H., Liu C.R., Du Y.J., Li L.S. (2022). Changes of macrophage and CD4 T cell in inflammatory response in type 1 diabetic mice. Sci. Rep..

[37] Kobayashi Y., Uehara S., Udagawa N., Takahashi N. (2016). Regulation of bone metabolism by Wnt signals. Int. J. Mol. Sci..

[38] Bullock W.A., Hoggatt A.M., Horan D.J., Lewis K.J., Yokota H., Hann S., Warman M.L., Sebastian A., Loots G.G., Pavalko F.M. (2019). Expression of a degradation-resistant beta-catenin mutant in osteocytes protects the skeleton from mechanodeprivation-induced bone wasting. J. Bone Miner. Res..

[39] Luan X., Lu Q., Jiang Y., Zhang S., Wang Q., Yuan H., Zhao W., Wang J., Wang X. (2012). Crystal structure of human RANKL complexed with its decoy receptor osteoprotegerin. J. Immunol..

[40] Xiang S., Zhu C., Zhou Y., Wu W., Zhang Y., Chen C., Wang F. (2024). Facile generation of neutralizing antibodies on tyrosine phosphorylated irs1 by epitope-directed elicitation. ACS Chem. Biol..

[41] Lee N.K., Sowa H., Hinoi E., Ferron M., Ahn J.D., Confavreux C., Dacquin R., Mee P.J., McKee M.D., Jung D.Y. (2007). Endocrine regulation of energy metabolism by the skeleton. Cell.

[42] Ye X., Yu R., Jiang F., Hou X., Wei L., Bao Y., Jia W. (2022). Osteocalcin and risks of incident diabetes and diabetic kidney disease: A 4.6-year prospective cohort study. Diabetes Care.

[43] Kanazawa I. (2015). Osteocalcin as a hormone regulating glucose metabolism. World J. Diabetes.

[44] Xu X., Liu S.Y., Liu H., Ru K., Jia Y.X., Wu Z.X., Liang S.J., Khan Z., Chen Z.H., Qian A.R. (2021). Piezo channels: Awesome mechanosensitive structures in cellular mechanotransduction and their role in bone. Int. J. Mol. Sci..

[45] Gudipaty S.A., Lindblom J., Loftus P.D., Redd M.J., Edes K., Davey C.F., Krishnegowda V., Rosenblatt J. (2017). Mechanical stretch triggers rapid epithelial cell division through Piezo1. Nature.

[46] Coste B., Mathur J., Schmidt M., Earley T.J., Ranade S., Petrus M.J., Dubin A.E., Patapoutian A. (2010). Piezo1 and Piezo2 are essential components of distinct mechanically activated cation channels. Science.

[47] Chen S., Li Z., Chen D., Cui H., Wang J., Li Z., Li X., Zheng Z., Zhan Z., Liu H. (2023). Piezo1-mediated mechanotransduction promotes entheseal pathological new bone formation in ankylosing spondylitis. Ann. Rheum. Dis..

[48] Sun W.J., Chi S.P., Li Y.H., Ling S.K., Tan Y.J., Xu Y.J., Jiang F., Li J.W., Liu C.Z., Zhong G.H. (2019). The mechanosensitive Piezo1 channel is required for bone formation. Elife.

[49] Liang T.Z., Jin Z.Y., Lin Y.J., Chen Z.Y., Li Y., Xu J.K., Yang F., Qin L. (2025). Targeting the central and peripheral nervous system to regulate bone homeostasis: Mechanisms and potential therapies. Mil. Med. Res..

