# Supplementary material for: Exercise Alleviates Osteoporosis and Hyperglycemia in Type 1 Diabetes Mellitus Mice via Piezo1-Mediated Mechanotransduction
Source: Biology (Basel). 2026 May 22;15(11):819. doi: 10.3390/biology15110819 (PMC13255602; doi:10.3390/biology15110819)
Supplement: Supplementary file 1 [file biology-15-00819-s001.zip › Original Images for Blots.pdf]

# Supplementary figures of western blot

**Table. S1 for Figure.1**

| Housekeeping proteins | Objective proteins | Image in paper |
|---|---|---|
|  | 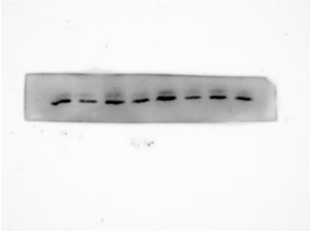 | 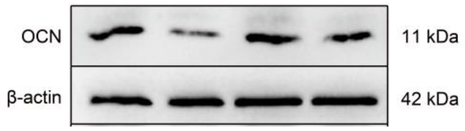 |
| 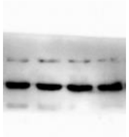 | 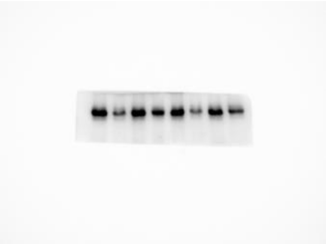 | 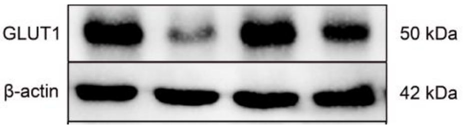 |
| 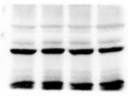 | 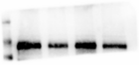 | 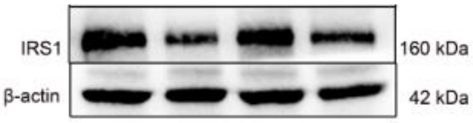 |
| 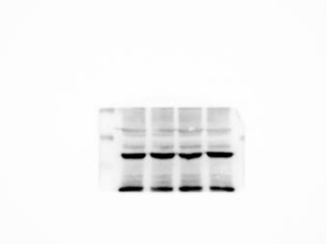 | 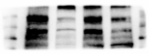 | 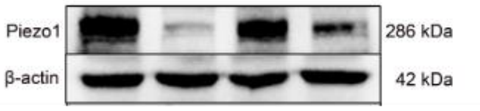 |
| 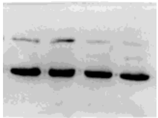 | 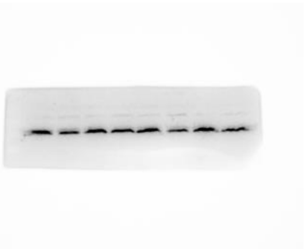 | 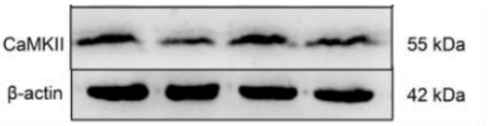 |

**Table. S2 for Figure.3**

| Housekeeping proteins | Objective proteins | Image in paper |
|---|---|---|
| 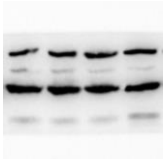 | 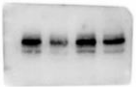 | 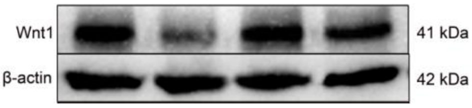 |
| 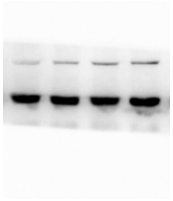 | 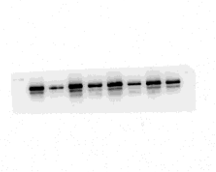 | 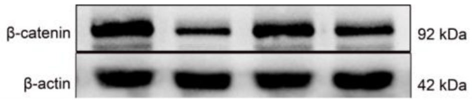 |
|  | 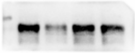 | 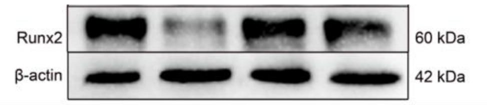 |
| 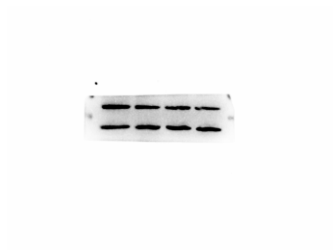 | 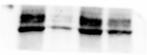 | 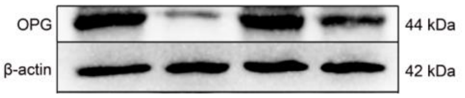 |
| 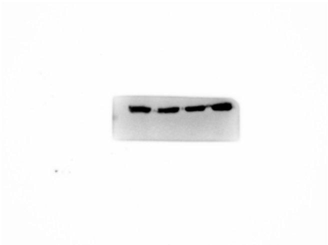 | 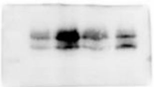 | 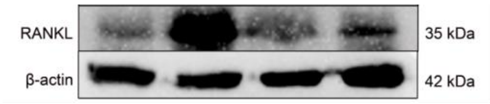 |

**Table. S3 for Figure.4**

| Housekeeping proteins | Objective proteins | Image in paper |
|---|---|---|
| 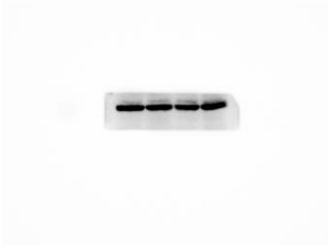 | 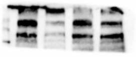 | 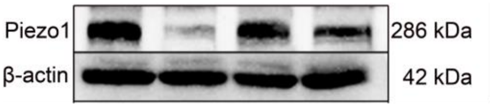 |
| 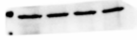 |  | 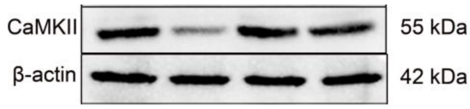 |
| 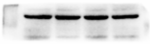 | 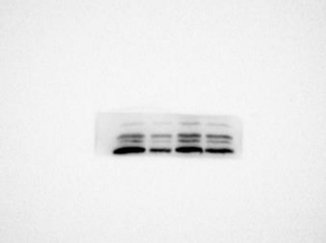 | 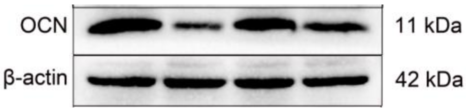 |
| 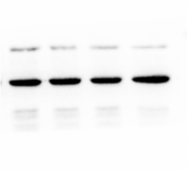 | 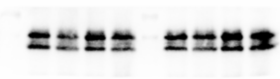 | 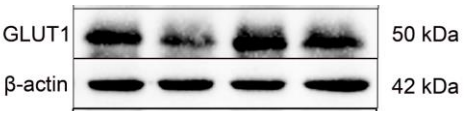 |
| 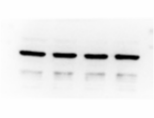 | 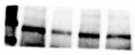 | 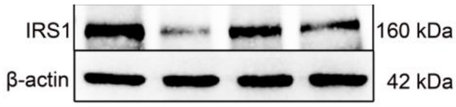 |
| 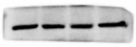 | 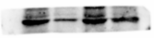 | 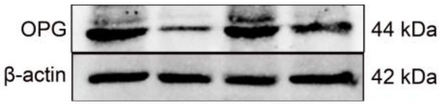 |

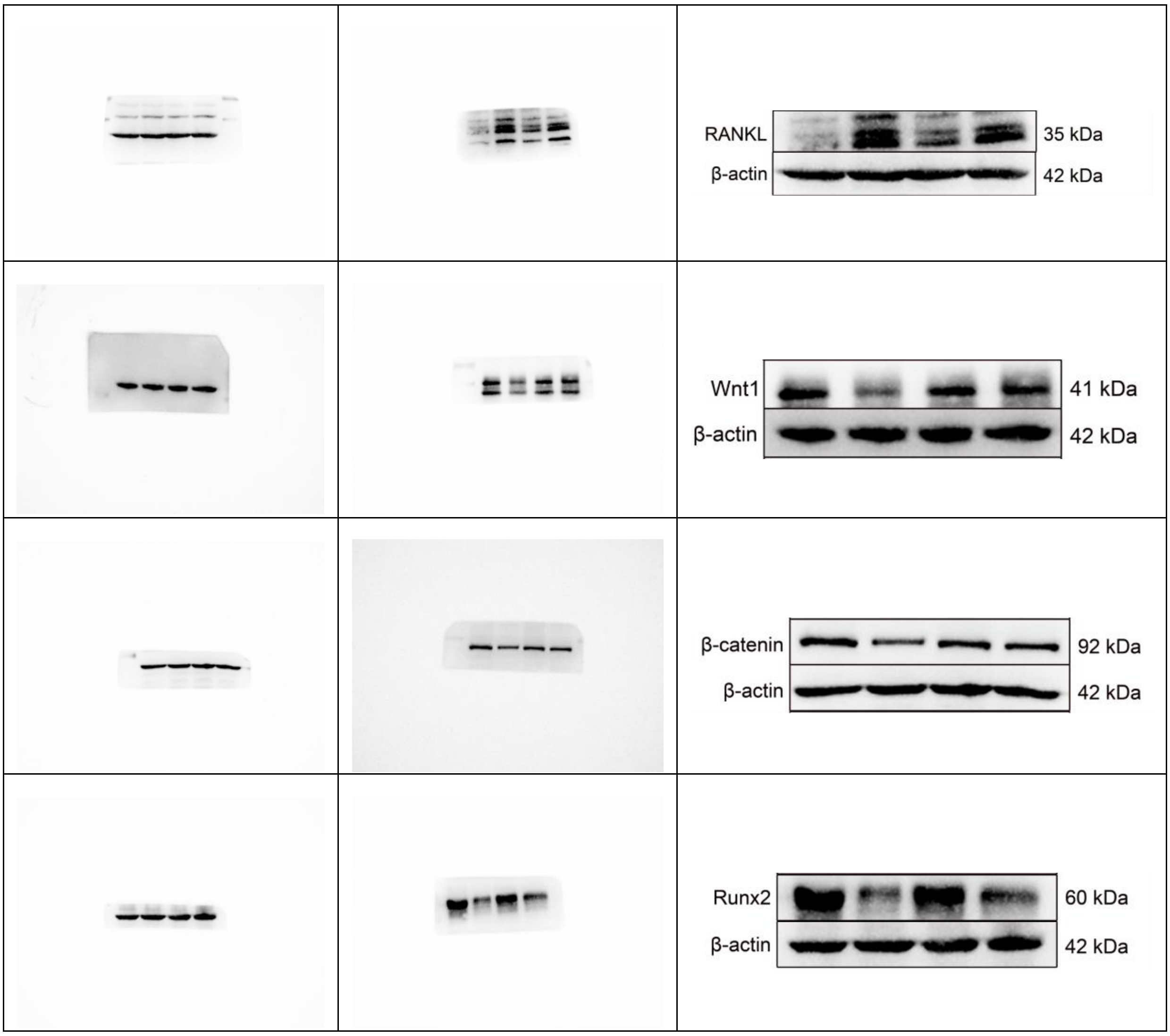
RANKL
35 kDa
β-actin
42 kDa
Wnt1
41 kDa
β-actin
42 kDa
β-catenin
92 kDa
β-actin
42 kDa
Runx2
60 kDa
β-actin
42 kDa

## Table. S4 for Figure.5

| Housekeeping proteins | Objective proteins | Image in paper |
|---|---|---|
| 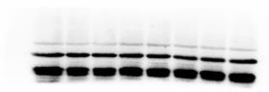 | 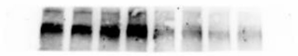 | 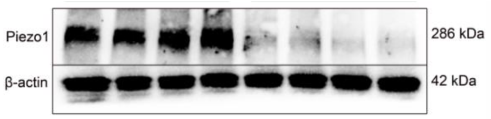 |

**Table. S5 for Figure.6**

| Housekeeping proteins | Objective proteins | Image in paper |
|---|---|---|
| 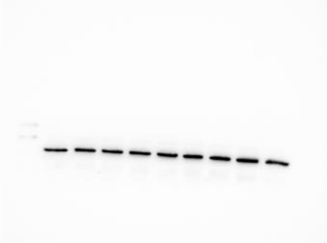 | 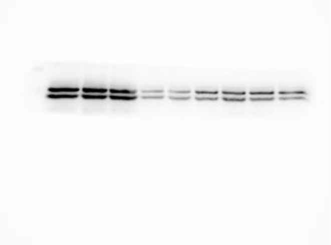 | 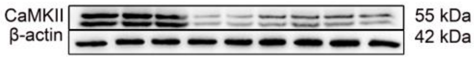 |
|  | 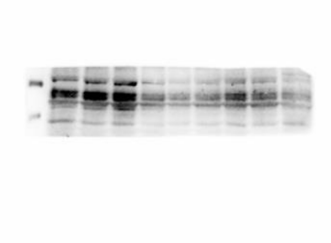 | 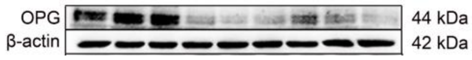 |
| 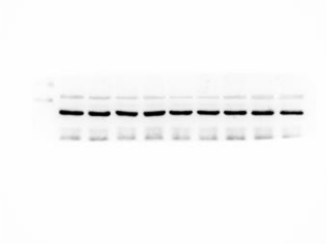 | 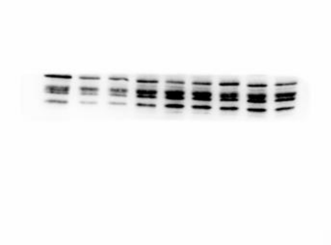 | 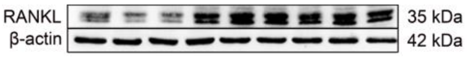 |
| 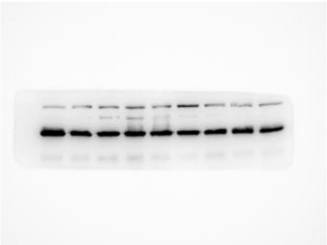 | 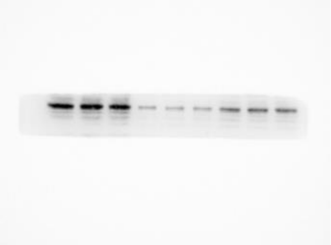 | 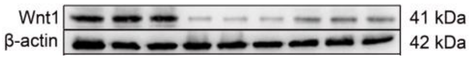 |
| 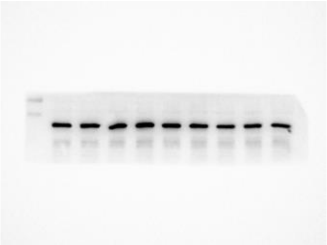 | 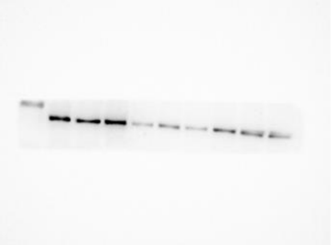 | 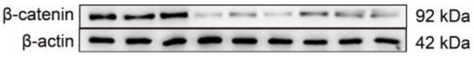 |
| 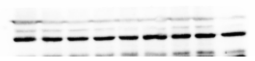 | 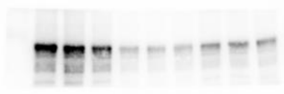 | 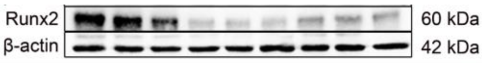 |

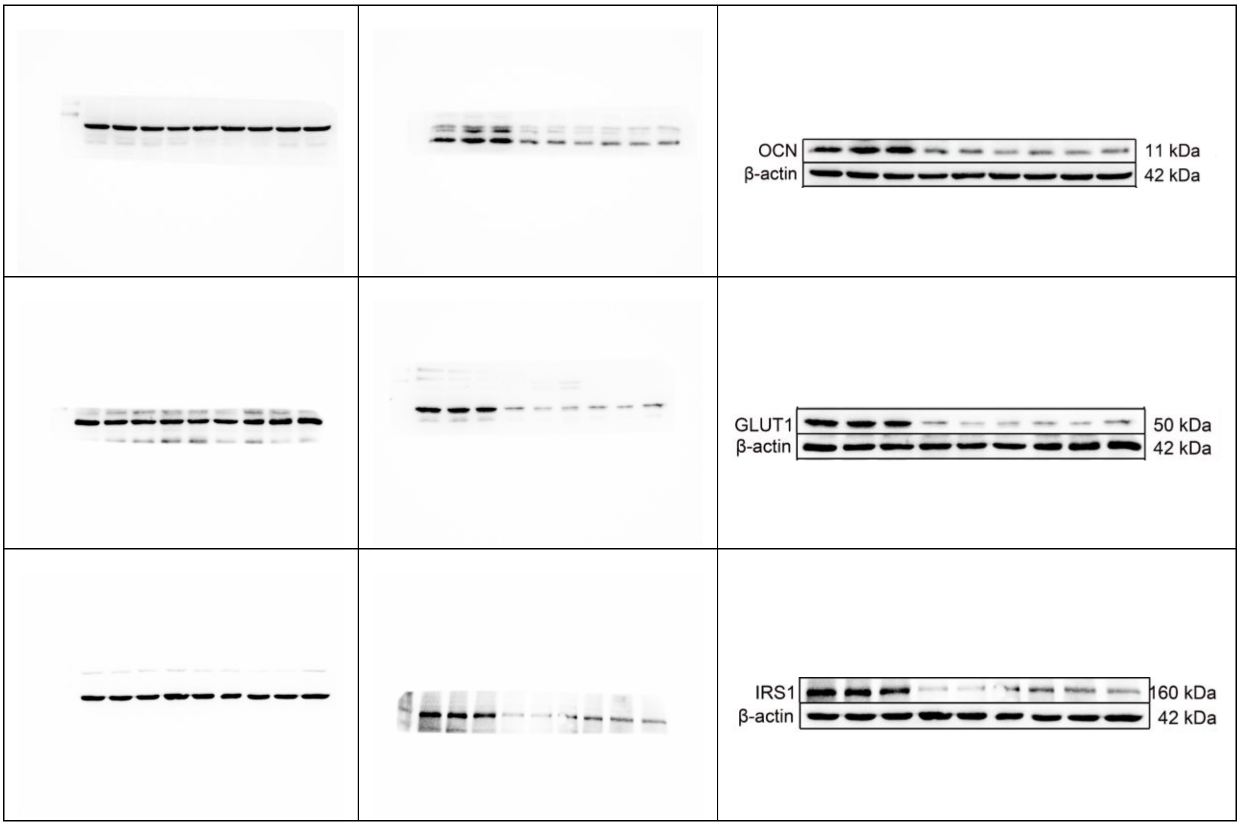
OCN
β-actin
11 kDa
42 kDa
GLUT1
β-actin
50 kDa
42 kDa
IRS1
β-actin
160 kDa
42 kDa

**Table. S6**

**Antibody Information**

| Antibody Name | Source | Clonality | Dilution Ratio | Manufacturer | RRID |
|---|---|---|---|---|---|
| β-actin | Rabbit | Polyclonal | 1:1000 | Servicebio | GB11002 |
| Goat Anti-Rabbit IgG (H+L) | Rabbit | Polyclonal | 1:5000 | zhuangzhibio | EK020 |
| Piezo1 | Rabbit | Polyclonal | 1:500 | proteintech | 15939-1-AP |
| CaMKII | Rabbit | Polyclonal | 1:1000 | ABclonal | A0198 |
| Wnt1 | Rabbit | Polyclonal | 1:1000 | proteintech | 27935-1-AP |
| β-catenin | Rabbit | Polyclonal | 1:1000 | proteintech | 66379-1-Ig |
| Runx2 | Rabbit | Polyclonal | 1:1000 | ABclonal | A2851 |
| OPG | Rabbit | Polyclonal | 1:1000 | Bioss | bs-20625R |
| RANKL | Rabbit | Polyclonal | 1:1000 | proteintech | 66610-1-Ig |
| OCN | Rabbit | Polyclonal | 1:1000 | ABclonal | A20800 |
| GLUT1 | Rabbit | Polyclonal | 1:1000 | proteintech | 66290-1-Ig |
| IRS1 | Rabbit | Polyclonal | 1:1000 | proteintech | 17509-1-AP |
