# Supplementary figures and images for: Exercise Alleviates Osteoporosis and Hyperglycemia in Type 1 Diabetes Mellitus Mice via Piezo1-Mediated Mechanotransduction
Source: Biology (Basel). 2026 May 22;15(11):819. doi: 10.3390/biology15110819 (PMC13255602; doi:10.3390/biology15110819)

Supplementary figures of TRAP

Table. S7 for Figure.2

|    |                                                                                                                                                                         |                                                                                                                                                                            |
|----|-------------------------------------------------------------------------------------------------------------------------------------------------------------------------|----------------------------------------------------------------------------------------------------------------------------------------------------------------------------|
| NC | 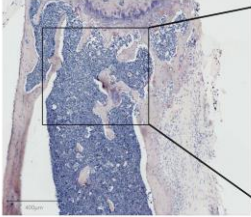 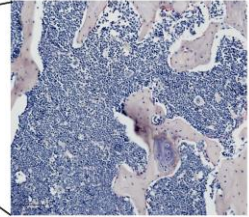     | 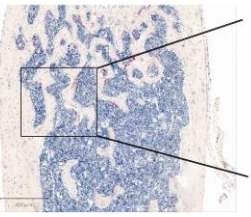 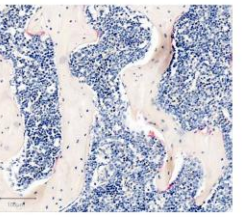     |
|    | 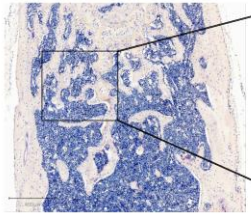 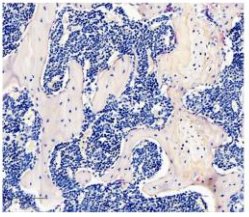     | 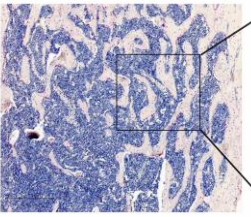 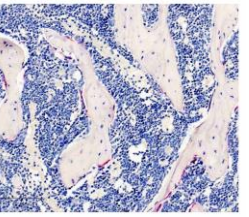     |
|    | 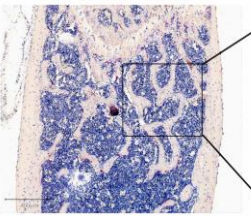 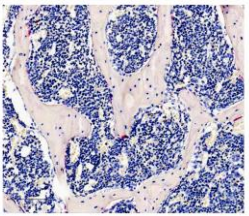   | 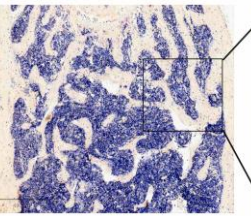 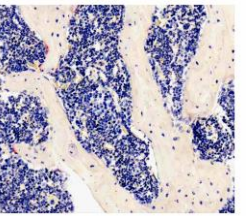   |
| DM | 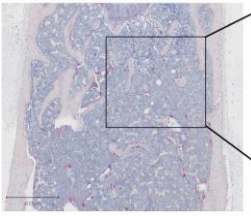 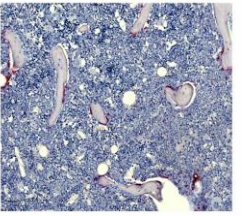 | 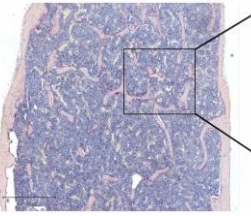 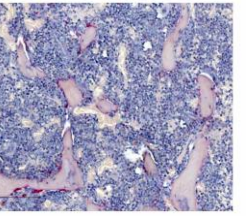 |
|    | 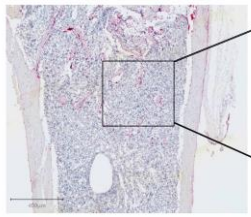 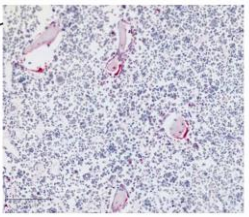 | 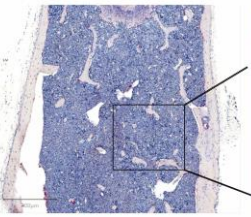 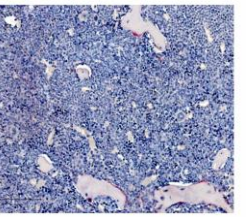 |
|    | 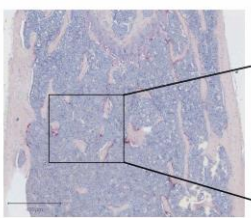 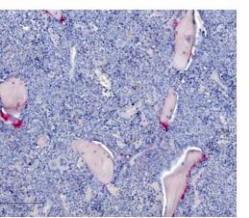 | 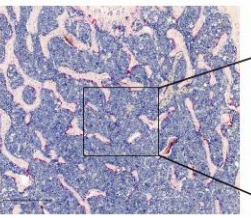 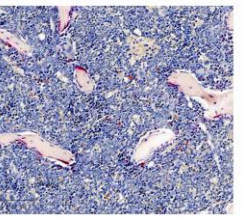 |

|                |                                                                                                                                                                         |                                                                                                                                                                            |
|----------------|-------------------------------------------------------------------------------------------------------------------------------------------------------------------------|----------------------------------------------------------------------------------------------------------------------------------------------------------------------------|
| DM<br>+<br>INS | 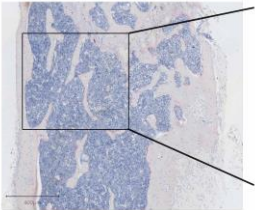 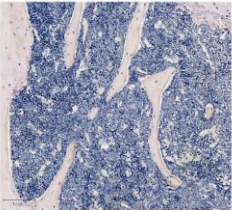     | 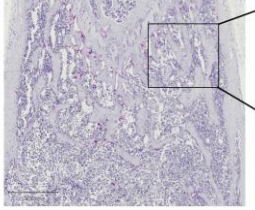 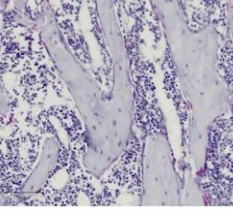     |
|                | 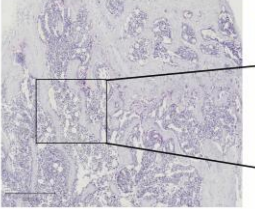 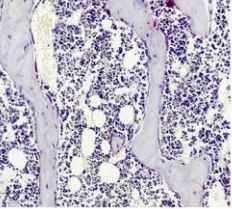     | 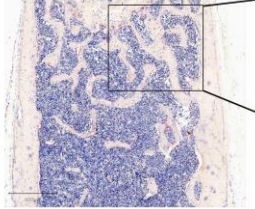 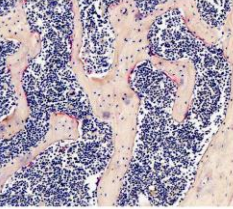     |
|                | 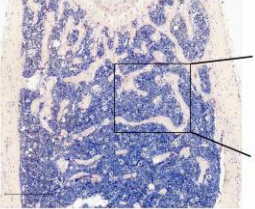 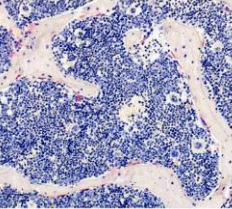     | 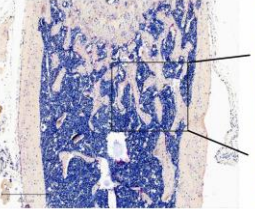 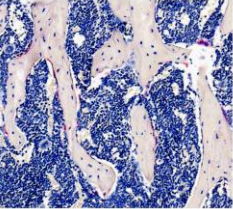     |
| DM<br>+<br>EX  | 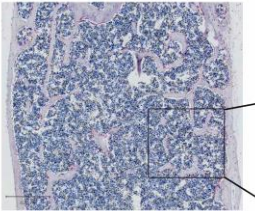 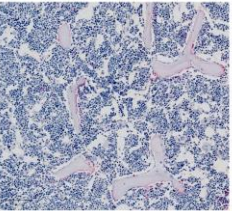 | 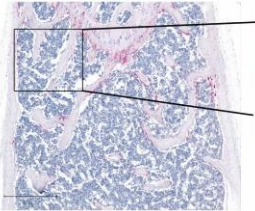 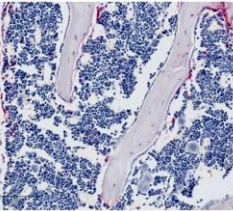 |
|                | 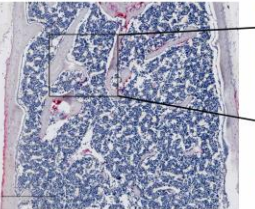 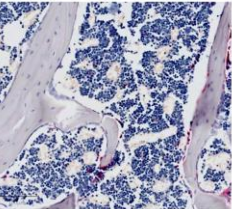 | 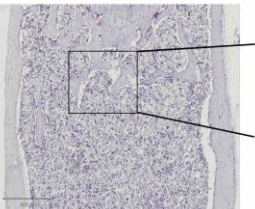 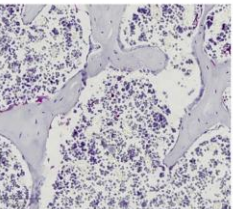 |
|                | 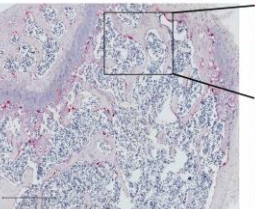 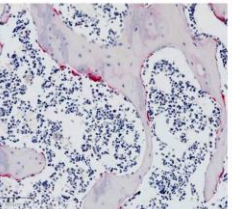 | 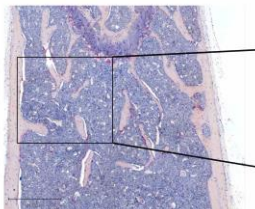 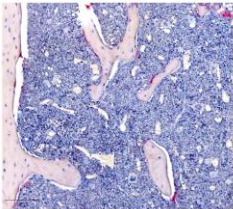 |

Supplement: Supplementary file 1 [file biology-15-00819-s001.zip › Supplementary figures of TRAP.pdf]
